# Spotlight on the Mechanism of Action of Semaglutide

**DOI:** 10.3390/cimb46120872

**Published:** 2024-12-23

**Authors:** Ilias Papakonstantinou, Konstantinos Tsioufis, Vasiliki Katsi

**Affiliations:** 1 4th Department of Internal Medicine, Evangelismos General Hospital, 10676 Athens, Greece; 2 1st Department of Cardiology, School of Medicine, National and Kapodistrian University of Athens, Hippokration General Hospital, 11527 Athens, Greece; ktsioufis@gmail.com

**Keywords:** semaglutide, adipose, browning, diabetes, obesity, inflammation, autophagy, infections, aging, sarcopenia

## Abstract

Initially intended to control blood glucose levels in patients with type 2 diabetes, semaglutide, a potent glucagon-like peptide 1 analogue, has been established as an effective weight loss treatment by controlling appetite. Integrating the latest clinical trials, semaglutide in patients with or without diabetes presents significant therapeutic efficacy in ameliorating cardiometabolic risk factors and physical functioning, independent of body weight reduction. Semaglutide may modulate adipose tissue browning, which enhances human metabolism and exhibits possible benefits in skeletal muscle degeneration, accelerated by obesity and ageing. This may be attributed to anti-inflammatory, mitochondrial biogenesis, antioxidant and autophagy-regulating effects. However, most of the supporting evidence on the mechanistic actions of semaglutide is preclinical, demonstrated in rodents and not actually confirmed in humans, therefore warranting caution in the interpretation. This article aims to explore potential innovative molecular mechanisms of semaglutide action in restoring the balance of several interlinking aspects of metabolism, pointing to distinct functions in inflammation and oxidative stress in insulin-sensitive musculoskeletal and adipose tissues. Moreover, possible applications in protection from infections and anti-aging properties are discussed. Semaglutide enhancement of the core molecular mechanisms involved in the progress of obesity and diabetes, although mostly preclinical, may provide a framework for future research applications in human diseases overall.

## 1. Introduction

The pathogenesis of obesity and type-2 diabetes mellitus (T2D) overlaps with shared interlinked molecular pathways involving insulin resistance, oxidative stress and inflammatory procedures, often driven by dysregulated interactions with the glucagon-like peptide 1 (GLP-1) and GLP-1 receptors (GLP-1R) system [1,2,3]. This receptor has been shown to affect multiple metabolic functions, including energy homeostasis, appetite, satiety, thermogenesis, inhibition of inflammation and protection against oxidative stress [1,4].

Semaglutide, a potent GLP-1R activator (GLP1-RA), is recommended by diabetes associations to improve glycemic control in patients with T2D and as a first-line therapy in established atherosclerotic cardiovascular disease (ASCVD) or multiple ASCVD risk factors to reduce the risk of major adverse cardiovascular events (MACE), i.e., myocardial infarction, stroke and cardiovascular death [5,6]. Semaglutide also showed a 20% reduction in MACE in overweight or obese adults with preexisting ASCVD but without diabetes, according to the Semaglutide Effects on Cardiovascular Outcomes in People with Overweight or Obesity (SELECT) trial [7,8]. Importantly, in 2021, semaglutide at a 2.4 mg injection was the first drug to be approved in the United States since 2014 for chronic weight management in obese and overweight patients [9]. Thereafter, semaglutide succeeded as the most media-friendly ‘miracle appetite suppressant’ anti-diabetic drug for weight loss. The body weight reduction in people with obesity with semaglutide 2.4 mg once weekly is approximately 15%, with simultaneous improvement in cardiometabolic risk factors and physical functioning [10,11]. This has been induced in persons who are overweight or obese with or without T2D [11,12]. From the newest sub-study of the SELECT trial, researchers found that compared to a placebo, patients treated with semaglutide 2.4 mg presented a significant reduction in non-ASCVD deaths, especially deaths from infections. These findings indicate that semaglutide or similar treatments may modify the risk of death due to multiple etiologies [13].

According to the traditional role in GLP-1 biology, semaglutide is an analog to GLP-1, a dipeptidyl peptidase-4 (DPP-4), degradation-resistant GLP-1RA with long-acting properties [14]. Binding and activation of GLP-1R lead to stimulation of pancreatic β-cells for a glucose-dependent insulin secretion, suppression of glucagon secretion, gastric emptying delay and reduction in food intake [14,15,16]. The regulation of appetite is also provided through the interaction with the hormone leptin [17]. GLP-1 is secreted in response to nutrients that pass through the gut from the enteroendocrine L cells, which are relatively low in the proximal small bowel and increase in the distal gut, reaching their highest density in the intestinal ileum and colon [18]. Beyond this, the enteroendocrine L cell function encompasses the sensing of inflammatory stimuli [19]. Other sources of GLP-1 are the pancreatic α-cells and, importantly, the neurons located in the brainstem [20].

The advent of recent research has shown that semaglutide not only potentiates insulin secretion and alleviates insulin resistance by inducing glucose transporter type 4 (GLUT4) in insulin-sensitive target tissues, mainly skeletal muscle and adipose tissue, but also presents exceptional anti-inflammatory and mitochondrial biogenesis regulating effects, mitigating oxidative stress [21,22]. Mainly, these effects are associated with inhibition of the pro-inflammatory transcription factor NF-κB, activation of key mitochondrial function elements like uncoupling protein 1 (UCP1) and activation of the energy and nutrient sensing adenosine monophosphate–activated protein kinase (AMPK) in association with sirtuin 1 (SIRT1), a nicotinamide adenine dinucleotide (NAD+)–dependent deacetylase well known for its role in redox hemostasis, inflammation and adipose tissue browning [1,22]. In particular, adipose tissue browning refers to the convergence of white to brown adipose tissue, which exerts a high metabolic rate by utilizing lipids to generate heat [23]. This enhanced thermogenic potential disposes of the excess calorie intake to generate additional thermal energy, elevating energy expenditure and promoting a negative energy balance [23]. Exactly in this way, semaglutide may interfere with low energy expenditure or impaired fat oxidation or both to favor body weight loss [24]. Beyond ameliorating inflammation and oxidative stress, semaglutide has demonstrated efficacy in regulating autophagy, a process that has recently gained attention as intracellular elimination of damaged cellular components or the regulation of cell apoptosis [25,26]. Autophagy demonstrates a significant mechanism for clearance of mitochondria, inducing healthy adipose tissue browning, and also in muscle aging and sarcopenia in obesity [27]. Because of mutual communication between skeletal muscle, adipose tissue and immune cells, there is a possible application of semaglutide in protection from infections [28] and tissue inflammation, as it acts on senescence-associated secretory phenotype (SASP), presenting anti-aging properties [29].

However, molecular mechanisms of how semaglutide may restore the balance of several interlinking aspects of metabolism to exert pleiotropic effects in obesity and diabetes complications are based mainly on preclinical data, while available evidence in humans appears to be lacking. For example, while semaglutide reduced the risk of clinically important kidney outcomes and death from cardiovascular causes in patients with T2D and chronic kidney disease [30], it is not known which mechanism(s) or processes from GLP-1/GLP-1R action delay the course of diabetic kidney disease, making these findings hypothesis-generating [31]. Under this aspect, we focused on conferring a ray of light on the advanced molecular mechanisms of action of semaglutide. This review was carried out from February to October 2024, and data were acquired from PubMed after observing and enrolling the most recent relevant to the term preclinical and clinical research and trials. No external funding was received. For clinical relevance and brevity, we will mainly focus on gaps in current knowledge on how semaglutide alters molecular mechanisms, especially in adipose tissue and skeletal muscle, and how it interacts with leptin, adipokines and myokines, enlightening possible benefits in the aging process and inflammation. Understanding the current situation may enhance future research and provide a framework for discussion on further perspectives in human diseases.

## 2. The Role of Semaglutide in Insulin Resistance

GLP-1 and insulin share interlinked common pathways [32]. At first, insulin is an anabolic hormone and under excess calorie consumption, suppresses lipolysis, promoting lipogenesis and fat storage in adipocytes [33]. Insulin binding to the insulin receptors (IR) triggers a downstream signaling cascade of phosphorylation of tyrosine residues on the insulin receptor substrates IRS-1 and IRS-2. The transmitted IRS signal is followed by phosphorylation of phosphatidylinositol 3-kinase (PI3K), protein kinase A (PKA), protein kinase C (PKC) and the mammalian target of rapamycin (mTOR), leading to GLUT4 translocation to cell membrane, thus inducing glucose uptake [34]. In obesity, pro-inflammatory cytokines, mainly interleukins IL-6, IL-1b and tumor necrosis factor α (TNF-α), are released and activate several serine kinases, including c-Jun N-terminal kinase (JNK) and extracellular signal-regulated kinase (ERK) [22,34,35]. JNK, an activator of inflammation and apoptosis, impairs insulin signaling by inhibition of IRS-1 signaling and GLUT-4 expression [22,36]. This metabolic inflexibility of significant decrease in tyrosine phosphorylation of IRS-1 is referred to as insulin resistance [37,38]. Besides directly inhibiting IRS-1, the underlying mechanism of insulin resistance by inflammatory mediators occurs through the activation of NF-κB, and inhibitor of nuclear factor kappa B kinase subunit β (IKKβ) [22,35,36,39].

To exert benefits in insulin action, GLP-1 binds to the G protein-coupled with the GLP-1R in pancreatic β-cells [40,41]. Then, the adenylate cyclase (AC) is activated. This leads to elevated levels of intracellular cyclic adenosine monophosphate (cAMP) and direct activation of PKA and cAMP regulated guanine nucleotide exchange factor 2 (EPAC2) [32,42]. Accordingly, the main action of semaglutide is to enhance glucose-dependent insulin biosynthesis and secretion through a cAMP-dependent PKA mechanism by conveying two insulin signaling pathways: the PI3K/PKA/mTOR in β-cells and the PI3K/AKT along with the AMPK/SIRT1 pathway [20].

First, the PI3K/PKA/mTOR pathway activates hypoxia-inducible factor 1 (HIF-1). This increases glucose-dependent insulin secretion via glycolytic genes activation in response to hypoxia and growth factors, which elevate the citric acid cycle and intracellular adenosine triphosphate (ATP) concentration, leading to closure of ATP-sensitive potassium channels depolarizing the cell membrane and then to calcium influx and exocytosis of pancreatic β-cells insulin-containing vesicles [43]. Furthermore, the mTOR-dependent HIF-1 activation by semaglutide counteracts DPP-4 degradation, prolonging the GLP-1R signal and maintaining insulin secretion [21].

Secondly, the PI3K/AKT promotes GLUT4 to the plasma membrane of muscle cells and adipocytes, enhancing glucose uptake and homeostasis, in an insulin-independent mechanism [21,44]. Semaglutide may mediate glucose transport through AMPK/SIRT1 because GLP-1RA can activate SIRT1 through cAMP-dependent PKA, and GLUT4 is a downstream of SIRT1 [45]. Of note, semaglutide may also improve insulin resistance, triggering upregulation of phosphorylated IRS-1 [46]. Moreover, the PI3K/AKT signaling pathway, activated by GLP-1, stimulates β-cell proliferation, reduces apoptosis and facilitates insulin secretion by regulating downstream effector molecules like Forkhead box O1 protein (FOXO1) [40,44]. Proliferation of islet β cells is also mediated by AMPK [21]. cAMP-response element binding protein (CREB), activated by GLP-1, may also confer anti-apoptotic actions [40]. The important action of semaglutide through GLP-1 enhancement is the inhibition of glucagon release from pancreatic α-cells, decreasing hepatic gluconeogenesis and maintaining glucose stability, especially postprandially [40,47].

## 3. Mechanisms in the Role of Semaglutide in Obesity

According to studies, semaglutide through GLP-1 enhances adipose tissue browning and the formation of new adipocytes and ameliorates dysfunctional adiposity in obesity [48]. Before proceeding to explanatory mechanisms, the function of adipose tissue is described.

### 3.1. Adipose Tissue Function

There are two main types of adipose tissues in the body, thermogenic brown adipose tissue (BAT) and energy-storing white adipose tissue (WAT) [49,50,51]. There is also beige adipose tissue (BeAT), with features of both brown and white adipocytes [50,51].

The BAT amount is very limited in adult humans, located in the supraclavicular region and in several depots surrounding large blood vessels above the diaphragm; this is known as perivascular adipose tissue (PVAT) [52]. While representing a small proportion of total adipose tissue, BAT can exert a sizable metabolic impact, promoting heat generation (thermogenesis) in the body by decomposing the contained fatty acids, stored as triglycerides (TG) in adipose tissue lipid droplets, for energy [53]. Released free fatty acids (FFAs) are then oxidized through uncoupling the mitochondrial electron transport chain from ATP synthesis by uncoupling protein 1 (UCP1) for heat production [54]. Therefore, the main characteristic of activated BAT is the high levels of mitochondria, which contain large numbers of UCP1, facilitating the lipolytic cycle [49,55]. One of the main mechanisms of BAT activation is mediated through β-adrenergic receptors [49]. From animal studies in mice, GLP-1 may enhance the function of BAT by modulating the activity of the sympathetic system through binding with GLP-1R in the central nervous system (CNS) [56]. This way, BAT may be associated with alleviating insulin resistance and beneficial effects in energy expenditure [57,58].

WAT is widely distributed and can also be further divided into visceral adipose tissue (VAT) and subcutaneous adipose tissue (SAT), located beneath the skin [59,60]. SAT is beneficial because it acts as a metabolic sink for additional lipid storage, which prevents the accumulation of VAT [59,61]. SAT is characterized by more numerous small adipocytes, and it is more insulin-sensitive than VAT due to a higher expression of IRS-1, protecting it from insulin resistance [60,62]. Actually, SAT is very important, rendering any incapacity to engage new adipose cells to store excess lipids in the SAT as a preamble to the hypertrophic (unhealthy) growth of WAT, which is further characterized by an accrual infiltration of macrophages, extensive fibrosis, limited angiogenesis and ongoing hypoxia [63]. SAT also serves as a barrier against dermal infections and external physical stress agents as well as preventing heat loss [48,64]. The main function of WAT is to store excess energy in the form of fatty acids (TGs) or to release glycerol and FFAs as energy reservoirs to fuel effector organs, skeletal muscle and BAT when required [49]. Because WAT mainly plays the role of storing energy, it presents a small number of mitochondria in the cell and contains unilocular lipid droplets [55]. VAT is also an essential endocrine organ and more structurally arranged than previously appreciated. VAT regulates whole body metabolism by the release of the hormone adiponectin, which redistributes fat deposited ectopically in the liver and muscle to the SAT depots, and by the hormone leptin, which reduces appetite and augments energy expenditure, controlling energy homeostasis [61,65]. However, VAT particularly displays a major role in insulin resistance and TNF, IL-6 mediated adipose tissue inflammation [66].

In BeAT, beige adipocytes share features of both brown and white adipocytes but are unique and distinct from them [55]. They have multilocular lipid droplets and compared to white adipocytes present higher mitochondrial content with higher levels of UCP1, sharing thermogenic and energy expenditure functions similar to that of classic BAT [55,67,68].

### 3.2. Fat Browning and Markers of Adipose Tissue Differentiation

GLP-1 controls the fate of preadipocytes, the differentiation to WAT or BAT and furthermore, the conversion of WAT through a browning process into beige adipocytes, a condition called adipose tissue browning [69,70]. This is determined by distinct transcription factors and proteins.

Major regulators of brown adipogenesis are PR domain-containing 16 (PRDM16), peroxisome proliferator activated receptor gamma coactivator 1 alpha (PGC1a), peroxisome proliferator activated receptor alpha (PPARα), fibroblast growth factor 21 (FGF21), protein fibronectin type III domain-containing protein 5 (FNDC5), irisin, FOXO1 and UCP1 [71,72,73,74].

On the other hand, signaling pathways and transcription factors implicated in WAT differentiation and lipid storage may include CCAAT enhancer binding protein alpha (C/EBPα), sterol regulatory element binding transcription factor 1 (SREBP1), peroxisome proliferator-activated receptor-γ (PPARγ) and SIRT1 [49,75,76]. C/EBPα and PPARγ induce and maintain the expression of key adipogenic genes (GLUT4 and ADIPOQ), which are necessary for normal adipocyte function [61,76].

### 3.3. Semaglutide Regulates WAT to BAT Conversion

According to interactions with the described transcription factors, semaglutide signal may promote the differentiation towards BAT and WAT browning, which seem to be a good way to increase mitochondrial biogenesis and fatty acid oxidation capacity, accelerating human metabolism and limiting weight gain [23,48,67]. However, the evidence on how this is realized is derived mainly from limited preclinical models, and the specific pathways involved need further research and exploration.

**Figure 1:**
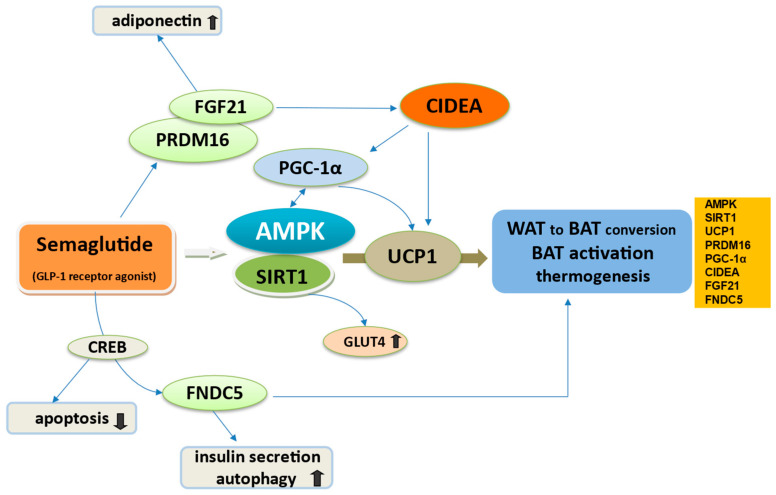
Semaglutide and transcriptional regulation of WAT to BAT conversion and BAT activation. Because of primitive experimental research in humans, data were acquired from reviews and from experimental evidence in rodents. Semaglutide activates the AMPK/SIRT1 axis which presents a crucial role for promoting the differentiation process into BAT by increasing the expression levels of UCP1. AMPK/SIRT1 activates PGC1α and via the transcriptional regulator PRDM16, the enzyme CIDEA. CIDEA controls the development of brown adipocytes in BAT and interacts with PGC1α to differentiate BeAT. Semaglutide may activate FGF21 produced by BAT which enhances the browning of WAT by increasing the expression of UCP1 and CIDEA. FGF21 exerts further anti-inflammatory effects by stimulation of adiponectin secretion. Semaglutide may induce FNDC5 expression in pancreatic β-cells via CREB, promoting the conversion of WAT to BAT, but also insulin secretion and autophagy while reducing apoptosis. Semaglutide may mediate glucose transport through AMPK/SIRT1 activation and GLUT4 upregulation. Upward arrows symbolize increase or upregulation, while downward arrows indicate decrease or downregulation of the mentioned mechanisms. Abbreviations: BAT, brown adipose tissue; BeAT, beige adipose tissue; WAT, white adipose tissue; UCP1, uncoupling protein 1; PRDM16, PR domain-containing 16; PGC1a, peroxisome proliferator activated receptor gamma coactivator 1 alpha; FGF21, fibroblast growth factor 21; FNDC5, protein fibronectin type III domain-containing protein 5; CREB, cAMP-response element binding protein; CIDEA, cell death-inducing DNA fragmentation factor-α-like effector A; GLUT4, glucose transporter type 4; AMPK, adenosine monophosphate–activated protein kinase; SIRT1, sirtuin 1.

The pivotal signaling pathway by which semaglutide through GLP-1/GLP-1R can enhance BAT thermogenesis-WAT browning is the master cellular energy regulation protein of the body, AMPK [77,78,79]. AMPK, through the activation of SΙRΤ1, another important mitochondrial activator, promotes the differentiation process into brown and beige, increasing the expression levels of UCP1 [49]. SIRT1 is found to be activated by semaglutide as determined by an experiment in mice [80] (Figure 1).

One way of functioning of the GLP-1/AMPK/SIRT1 system is CNS AMPK/SIRT1 activation in the hypothalamic ventromedial nucleus by GLP-1R stimulation, in addition to an increase in sympathetic nervous system activity in β3-adrenergic receptors via hypothalamic AMPK/SIRT1 activity [78,81,82]. This was noted in rodents and humans [82].

Together with the CNS action, the GLP-1/AMPK/SIRT1 axis enhances BAT activation via transcriptional regulator PRDM16 and activating PGC1α [67,83]. Among them, the BAT programmer coactivator PRDM16 leads to the selective induction of BAT genes like cell death-inducing DNA fragmentation factor-α-like effector A (CIDEA), an outstanding protein that controls the development of brown adipocytes in BAT while inhibiting typical WAT markers [49,73]. Increased expression of the lipid droplet surface protein CIDEA in the SAT may increase adipocyte size by increasing triglyceride storage [61]. PGC-1α is the master transcriptional regulator of genes involved in mitochondrial biogenesis and oxidative metabolism and is particularly expressed in skeletal muscle and adipose tissue [83]. In adipose tissue along with PPAR-γ and alpha (PPAR-α), PGC-1α participates in BAT activation and by SIRT1 deacetylation regulates mitochondrial biogenesis and enhances mitochondrial fatty acid oxidation [84,85]. Notably, CIDEA interacts with PGC1α to differentiate BeAT. In skeletal muscle, PGC-1α regulates muscle fiber type, averts muscle atrophy and regenerates muscle damage in obesity by activating AMPK and SIRT1 [83].

Another factor is FGF21 produced by BAT and BeAT, which enhances the browning of WAT and increases fatty acid oxidation by increasing the expression of UCP1 and CIDEA to promote thermogenesis and improve energy balance [86,87,88]. FGF21 exerts further anti-inflammatory effects by stimulation of adiponectin, which can enhance lipid and glucose metabolism and therefore mitigate the risk of obesity, diabetes and ASCVD [89]. According to a study in mice, semaglutide treatment may stimulate FGF21 in mouse liver and improve FGF21 sensitivity, suggesting an important role of the GLP-1/FGF21 axis in mediating beneficial effects in obesity [90].

The latest research pointed out that irisin, a hormone produced by skeletal muscle in response to exercise, derived from its precursor FNDC5, is crucially involved in the transformation of WAT to BAT and BAT-like development of WAT [91]. Irisin enhances the polarization of anti-inflammatory variants of macrophages to the M2 subtype in adipose tissue. This presents a beneficial metabolic effect by the secretion of exosomes containing microRNA (miRNA-690) to stimulate insulin sensitivity and the PPAR-γ-related anti-inflammatory system along with nuclear factor (erythroid-derived 2)-like 2 (NRF2)-related antioxidant genes [92]. GLP-1 induces FNDC5 expression in pancreatic β-cells via CREB promoting the conversion of WAT to BAT and thereby enhancing energy metabolism [1]. Notably, GLP-1 and irisin are synthesized within the pancreatic islets in α- and β-cells, respectively, and share comparable pleiotropic effects, activating similar intracellular pathways [93]. It is suggested that activating the FNDC5 gene not only stimulates glucose-dependent insulin secretion but also induces autophagy [94]. Finally, in a study on mice to elaborate the subject of interactions between adipocytes and semaglutide, Martins et al. found that semaglutide enhanced UCP1 multiloculation in the SAT of obese mice [95].

### 3.4. Action of Semaglutide in Dysfunctional Adiposity

In obesity, biological transformations occur, consisting of the failure of adipose precursor cells to form new adipocytes and the expansion of visceral WAT towards abnormal development and remodeling [76,96,97]. This contributes to adipose tissue dysfunction, whereas WAT inflammation and oxidative stress supervene, promoting the release of pro-inflammatory adipokines, leptin and adiponectin, along with enormous levels of cytokines (IL-6, IL-1b, TNF-α) and NF-κB activation, all ending in a cycle of sustained systemic insulin-resistant state [36,98,99,100]. This may be attributed to reduced AMPK activity in metabolic tissues in obesity [77]. Downregulation of adiponectin in the dysfunctional WAT restrain lipoprotein lipase (LPL), the luminal protein responsible for hydrolyzing plasma TGs and releasing FFAs for uptake into tissues escalating the storage of TGs in skeletal muscles and insulin signaling impairment [100,101,102,103]. LPL is also inhibited by angiopoietin-like 4 (ANGPTL4), a dominant regulator of LPL activity. This occurs in the fasting state where ANGPTL4 is upregulated in WAT while being inhibited in muscle and BAT [104].

The pro-inflammatory cytokines either inhibit adipogenesis or the ability of pre-adipocytes to mature into functional lipid-enriched adipocytes or predispose to the differentiation of pre-adipocytes into macrophage-like cells [105]. Particular involvement in these inflammatory processes in obesity present neutrophils and macrophages, which produce, besides the pro-inflammatory cytokines, a potent chemokine that promotes neutrophil recruitment and adhesion, the CXC motif chemokine ligand 2 (Cxcl2) [106,107]. Overexpression of Cxcl2 causes increased inflammation in WAT and to the cardiovascular system [106,108]. Moreover, neutrophils and macrophages contain the S100 protein family of two calcium-binding proteins, S100A8 and S100A9, which are involved in inflammatory processes and the molecular evolution of obesity [109,110].

One of semaglutide’s specific actions is to significantly reduce macrophage activity in adipose tissue and decrease the levels of inflammatory mediators IL-6, TNF-a and NF-kB together with JNK activation [22,35]. This may be mediated through GLP-1/AMPK increase in SIRT1 deacetylation of NF-κB [80]. A particular capability of semaglutide is to exert anti-inflammatory effects, possibly by inhibiting the expression of key factors in WAT inflammation S100a8, S100a9 and Cxcl2 in neutrophils [110,111]. Of interest, in obese mice, semaglutide can effectively reduce WAT through the regulation of lipid uptake, lipid storage and lipolysis by reducing the activity of ANGPTL4 and LPL, which are involved in adipocytes hypertrophy [112].

## 4. The Role of Semaglutide in Appetite Regulation

### 4.1. Semaglutide and Direct GLP-1 Regulation of Appetite in the Brain

Widely distributed in the brain CNS, GLP-1Rs display a crucial role in the regulation of hunger, satiety and feeding behavior to reduce energy intake [113,114]. Semaglutide presents a pronounced weight-lowering effect, mediated by direct GLP-1 stimulation of the anorexigenic (satiety inducers) proopiomelanocortin (POMC) and cocaine- and amphetamine-regulated transcript (CART) neurons along with indirect inhibition of orexigenic neurons (appetite inducers) neuropeptide Y (NPY) and agouti-related peptide (AgRP), located in the arcuate nucleus (ARC) in the hypothalamus, which is vital in the regulation of appetite and energy balance [114,115,116]. These neurons present functional connectivity with the nucleus of the solitary tract (NTS), which transmits peripheral signals from changes in nutrients, hormones, and neuropeptides to the hypothalamus [117]. An important mediator in the process of reduced feelings of hunger is the hormone leptin, which, in interaction with GLP-1, regulates satiety and controls reward and aversion, suppressing hunger by inhibiting the appetite inducers AgRP/NPY neurons, while stimulating the satiety inducers POMC neurons [17,118,119]. Semaglutide also increased the expression levels of SIRT1 and GLUT4 in the hippocampus of 3xTg mice and through the GLP-1R/SIRT1/GLUT4 pathway can mediate glucose transport, thereby improving glucose metabolism in the brain [120]. In addition to this, studies in animal models have suggested that activating the GLP-1Rs in the brain improves neuroinflammation and neurogenesis and confers neuroprotection [46,80,120,121].

Nevertheless, the mechanisms by which semaglutide exerts anorexigenic effects in these complex neuroanatomical regions of the brain that receive inputs from the limbic system are not completely understood [122]. A key component of the limbic system, the amygdala, displays a central role in emotional and motivational processing of feeding, along with emotional and survival behaviors including responses to predators and noxious stimuli [122]. Researchers have also shown that semaglutide impacts the reward and punishment systems in the brains of mice, particularly in the limbic system [123]. It would be therefore reasonable when prescribing or taking semaglutide or other GLP-1R GLP-1RAs to evoke how GLP-1RAs work; they do affect the brain.

### 4.2. The Input of Semaglutide in Leptin Signaling

Notably, leptin may serve as a crucial biological signal for GLP-1, working in synergy, to decrease food intake and body weight and increase energy expenditure [124]. When the fat cells increase, leptin, predominantly derived by SAT, binds to leptin receptors (Lep-R) located on AgRP neurons in the hypothalamus and on pre-proglucagon (PPG) neurons in the NTS to converge the GLP-1 signal and to exert anorexigenic effects, appetite suppression and satiety in addition to increased thermogenesis and energy expenditure to stimulate weight loss [17,125]. In vital metabolic tissues such as skeletal muscle, leptin is essential for preserving glucose balance by improving glucose absorption, glycogen formation and the oxidation of glucose and fatty acids [126]. In adipose tissue, leptin counteracts insulin’s action by inhibiting insulin-induced IRS-1 activation and PI3K binding [126]. Accordingly, leptin increases insulin sensitivity, specifically insulin-stimulated glucose utilization or glucose oxidation in skeletal muscle and BAT, but this is blunted in WAT [126]. Individuals with obesity and T2D present leptin resistance and higher circulating levels that drastically reduce the signaling of leptin in POMC neurons and the anorexigenic activity in the ARC [127,128].

Leptin signaling is based on the autophosphorylation of the Lep-R, triggering the Janus tyrosine kinase (JAK), the signal transducer and activator of transcription (STAT), PI3K, IRS and AMPK pathways [116]. The activation of each of these very important pathways in energy homeostasis contributes to the anorexigenic effects of leptin [125]. As was shown from studies in high-fat diet fed mice, in obesity, important mediators in the observed leptin resistance in AgRP neurons are JNK, NF-kB, suppressor of cytokine signaling 3 (SOSC3), protein tyrosine phosphatase 1B (PTP1B, also known as polypyrimidine tract binding protein 1), and PKC [127,128]. Experiments in mice indicated that SOCS3 along with PTP1B can negatively regulate the upstream activator JAK/STAT pathway suppressing it; this effectively dampens leptin signaling [116,125,129].

Leptin has also been proposed, mainly based on data from preclinical studies, to influence sympathetic nervous system activity promoting lipid mobilization in WAT and inducing thermogenesis in BAT through the increased expression of UCP1 [116]. This may be mediated mainly by activating proopiomelanocortin neurons, which, in turn, activate hypothalamic melanocortin 4 receptors (MC4R) that receive projections of POMC fibers, responsible for the anorexigenic activity [130,131]. Notably the effects of leptin on the sympathetic nervous system have not yet been replicated in humans, possibly showcasing a difference between leptin biology in animals and humans [124].

Semaglutide by GLP-1-leptin interaction may steer intracellular signaling involving the anorexic nervous system, leptin-sensitive JAK/STAT signaling and inhibition of their suppressor PTP1B/SOCS3 pathway, which counteracts leptin resistance [116]. Martins et al., in experiments in obese mice, showed changes in anorexigenic hormones with POMC/MC4R lessened activation and orexigenic NPY/AgRP increased activation, accompanied by leptin resistance and increased *Socs3* gene expression, blocking the central action of leptin [131]. Semaglutide treatment restored GLP-1 levels, regulated *Socs3* expression in ARC and improved leptin sensitivity and the hypothalamic anorexigenic signaling (POMC/MC4R) for obesity control in these obese mice [131].

## 5. Semaglutide and Oxidative Stress

**Figure 2:**
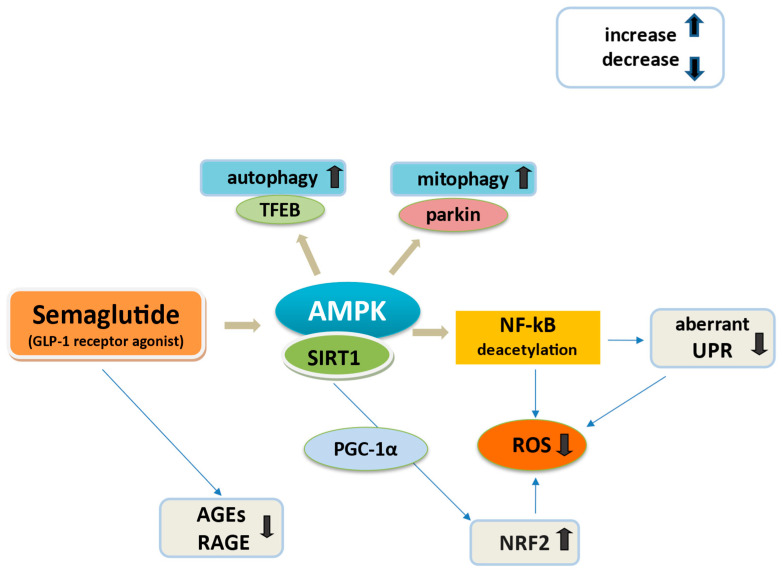
Possible mechanisms of semaglutide on oxidative stress and inflammation. Semaglutide activates AMPK/SIRT1 to deacetylate the NF-kB and possibly interacts with AGE/RAGEs to mediate critical anti- inflammatory responses and ROS reduction. Also PGC1α activated by SIRT1 regulates factors like NRF2 to diminish oxidative stress, aberrant UPR and ROS. In addition, semaglutide may promote autophagy and mitophagy through AMPK/TFEB and AMPK/parkin activation respectively. Upward arrows symbolize increase or upregulation, while downward arrows indicate decrease or downregulation of the mentioned mechanisms. Abbreviations: AMPK, adenosine monophosphate–activated protein kinase; SIRT1, sirtuin 1; NF-kB, nuclear factor kB; TFEB, transcription factor EB; UPR, unfolded protein response; ROS, reactive oxygen species; NRF2, nuclear factor (erythroid-derived 2)-like 2; AGEs, advanced glycation end-products; RAGE, AGE receptors.

Semaglutide through GLP-1R activation appears to regulate mitochondrial function and oxidative stress, although the mechanism by which this occurs has not been well elucidated. Proposed mechanisms are depicted in Figure 2.

### 5.1. Drivers of Mitochondrial and Endoplasmic Reticulum Oxidative Stress

Oxidative stress and increased reactive oxygen species (ROS) display an important role in the pathogenesis of insulin resistance in diabetes and obesity [4,132,133]. Ultimately, the impaired ability to switch from lipid to carbohydrate oxidation under insulin-stimulated conditions leads to mitochondrial damage and dysfunction [58]. Mitochondria, found in all nucleated human cells [134], present major sources of ROS, which are generated from nicotinamide adenine dinucleotide phosphate (NADPH) oxidase (NOX) enzymes [4,135,136]. Mitochondrial dysfunction, characterized by impaired β-oxidation capacity and increased ROS formation, fuels the aberrant ROS production [137]. ROS diminishes proteasomal and autophagy processes, accompanied by the detrimental accumulation of protein aggregates and damaged mitochondria [138,139]. Mitochondrial oxidative stress is further accompanied by endoplasmic reticulum (ER) stress, referred to as a homeostatic imbalance within the ER, caused by external stimulus or changes in the intracellular environment, which accelerate the accumulation of unfolded or misfolded proteins, all of which crucially exhibit cell dysfunction and the development of metabolic and aging-associated illnesses [140]. Protective cellular major antioxidant systems against cytotoxic effects are mitochondrial UCP and unfolded protein response (UPR) [128,135]. Although proper UPR acts as protective against misfolding, enhancing the ER folding capacity and misfolded proteins degradation, excessive or persistent UPR can trigger cellular damage, which leads to apoptosis [135]. ER stress activates the JNK and NF-κB pathway followed by UPR activation of the unfolded protein response [141]. Notably, insulin is formed as pro-insulin in the ER and further processed in the secretory vesicles of pancreatic β-cells to become bioactive [32,142].

Drivers of excessive ROS production along with mitochondrial and ER oxidative stress are WAT chronic inflammation, insulin resistance in obesity and advanced glycation end products (AGEs) produced in T2D [140,143,144]. In over-accumulated WAT, fatty acid by-products, mainly diacylglycerol (DAG), are metabolized through β-oxidation in the tricarboxylic acid (TCA) cycle, and under the DAG stimulation of PKC, the NADPH oxidase is directly activated to generate excess ROS [145]. ROS can inhibit IRS1 activation, thereby impairing insulin signaling and aggravating insulin resistance [35,145,146,147]. Additionally, AGEs, which act via AGE receptors (RAGE), present a key promotive factor in chronic inflammation [148]. They induce ROS overproduction and sustained activation of NF-κB, initiating pro-inflammatory responses, exacerbating cell damage and further advancing inflammation [149,150,151].

### 5.2. Semaglutide and Oxidative Stress Regulation

The improvement of inflammation and oxidative stress by semaglutide seems to be related both by modulating SIRT1/NRF2 signaling and suppressing the activated JNK/NF-κB pathways [80,152]. SIRT1 demonstrates the potential to some extent to activate the NRF2 pathway to alleviate oxidative stress by upregulation of antioxidant enzymes like superoxide dismutase (SOD) [4]. In addition, PGC1α activated by SIRT1 regulates factors like NRF2 to mitigate oxidative stress [137].

As previously mentioned, semaglutide reduced levels of ROS in an experiment in mice; this occurred in cardiac tissue and serum [110]. Semaglutide also significantly reduces macrophage activity in adipose tissue and decreases the levels of inflammatory mediators NF-kB together with JNK activation [22,35,153]. This may be mediated through GLP-1/AMPK increase in SIRT1 deacetylation of NF-κB by employing NAD+, significantly diminishing ROS and possible aberrant UPR activation of the unfolded protein response [135,137]. In diabetic mice, semaglutide was shown to ameliorate oxidative stress, elevating SIRT1/AMPK, hindering NF-κB activation and restoring connexin 43 (Cx43) expression, a key protein in ventricular myocardial gap junctions, which maintains electrical signal conduction between myocardial cells [154]. Semaglutide may possibly increase the expression of ubiquitin ligase parkin, leading to ubiquitination of mitochondrial proteins, effectively marking them for removal through mitophagy activation [137]. The latter represents a cargo-specific form of autophagy selectively targeting the degradation of dysfunctional, damaged and hence potentially cytotoxic mitochondria within a cell [137,155]. AMPK may be directly amenable to activation by semaglutide, and mitophagy may be initiated by AMPK phosphorylation of parkin [77,156,157]. AMPK is also critical for autophagy, phosphorylating a critical regulator of lysosomal biosynthesis, the transcription factor EB (TFEB) [77,157].

Recently, in the study of Martins et al. semaglutide enhanced UCP1 and lessened ER stress genes, presenting a significant anti-inflammatory beneficial effect beyond weight loss, in obese mice [95]. In addition, because GLP-1 inhibits the AGEs-induced RAGE gene expression and ROS generation in T2D [158], it may be hypothesized that semaglutide could reduce ROS production through AGE-RAGE mediation [159].

## 6. Semaglutide and Body Composition

Semaglutide demonstrated positive effects on body composition [47] with experimental evidence in obese mice showing body weight reduction and an optimal increase in muscle mass [160]. To demonstrate the molecular role of semaglutide in skeletal muscle homeostasis, first are described the reported weight loss effects of semaglutide and the mechanisms of sarcopenia induced by obesity and diabetes.

### 6.1. Semaglutide Weight Loss Effects and Impact on Muscle Mass

To interpret the changes in body composition, it is important to understand that body composition measurement divides the body into compartments according to different physiological properties, which commonly include fat mass (FM), the mass of all adipose tissue; fat free mass (FFM), the total body mass minus total fat mass; lean body mass (LBM), the fat free mass minus total bone mass; and skeletal muscle mass (SKM), the lean body mass minus connective tissue, skin and other organs. Other compartments include bone mineral content and total body water [161].

Fat loss during GLP-1RA treatment is associated with varying degrees of muscle mass loss [24,162]. Semaglutide particularly displays potential for weight loss ranging from almost 0% to 40% primarily through fat mass reduction. While a weight loss not exceeding 40% of the total body weight loss is considered healthy, semaglutide-induced decline in lean and skeletal mass may occur, raising concerns for notable reductions in lean mass, especially in trials with a larger number of patients [163]. Others showed that semaglutide presents weight reduction, attributed to fat mass, with an apparent positive impact on preserving muscle mass and muscle strength. These changes occurred rapidly and without relevant modifications to lifestyle and nutritional behaviors [47,164]. Observations in human trials reported that semaglutide effectively reduced body fat while maintaining muscle mass in obese T2D patients [165,166]. Further, experiments in obese mice indicated that semaglutide reversed muscle loss by reducing inflammatory response and insulin resistance, which was shown in significantly improved muscle fiber structure and increased number of mitochondria [24]. It should be underlined that the beneficial effects resulting from even a 5–10% weight loss can be invalidated if there is a concomitant exaggerated loss of lean mass. This may be attributed to muscle strength loss and reduction in physical efficiency along with the accompanied risk of developing insulin resistance if lean mass is diminished [164]. Therefore, in patients receiving GLP-1-based therapies, considering possible statistically significant reduction in muscle tissue, it would seem highly advisable to implement targeted physical exercise to preserve and potentially enhance muscle mass [47,167].

### 6.2. Skeletal Muscle Malfunction in Obesity and Diabetes

Skeletal muscle is an important participant in insulin action, stimulating GLUT4 glucose transporters on myocyte membranes and thereby increasing overall glucose uptake [168]. Moreover, skeletal muscle is the main site of amino acid metabolism, with an essential role in protein synthesis, muscle structure and the maintenance of muscle function. A decrease in their level directly affects protein synthesis, which leads to a decrease in muscle mass [169]. Patients with obesity and/or T2D often experience a decline in SKM coupled with functional deterioration and muscle atrophy, a condition called sarcopenic obesity (SOB) [170]. Sarcopenia is also age-associated but may be exacerbated by obesity, leading to higher disability, frailty, morbidity and mortality rates [171]. In addition, T2D patients are prone to losing SKM more rapidly than non-diabetic people, thus becoming more susceptible to insulin resistance than the general population. [171].

In obesity, excessive production of and disturbed capacity to store lipids in adipose tissue favor the infusion of lipids (DAGs and ceramides) ectopically in skeletal muscle both within and between muscle cells [170]. Increased DAG levels inhibit PKC, leading to defective activation of IRS-1/PI3K/AKT insulin signaling, downregulation of GLUT4 and forthcoming insulin resistance [170,172]. This has been reported in animal models and in humans [147]. Meanwhile, DAG diminishes the concentration of adiponectin in skeletal muscle, further promoting the accumulation of intramuscular lipids, providing a lipotoxic environment that leads to cell damage [34,146,147]. Furthermore, ROS overproduction in myoblasts causes accumulation of S100B, which stimulates NF-κB activity, resulting in upregulation of the Ying Yang 1 (YY1) axis and YY1-dependent inhibition of microRNA-133, a promyogenic and anti-adipogenic microRNA, ultimately leading to the transition of myoblasts into adipocytes [110,173,174]. As known, semaglutide, may inhibit the expression of the S100 family proteins to exert myogenic effects [110].

Key characteristics of SOB include the establishment of mechanisms for the development of muscle atrophy [171]. Experiments have shown that old sedentary rat muscles exhibit elevated levels of the atrophy genes (atrogenes) atrogin-1 and muscle ubiquitin ligase muscle ring finger1 (MuRF-1), both of which are targets of the FOXO family of transcription factors [175]. Increased ROS, activated NF-κB and insulin resistance enhance the FOXO transcription and upregulate the expression of Atrogin1 and MuRF1 [170,173]. Subsequently, atrogenes trigger the ubiquitin–proteasome system (UPS), the central proteolytic machinery of mammalian cells and moreover the lysosome-autophagy system, which significantly contribute to disturbed mitochondrial fission as the first step of mitophagy and muscle remodeling, causing severe muscle atrophy [176]. Another important regulator of muscle metabolism is AMPK, SIRT1 and PGC-1α signaling, displaying a crucial role in the prevention of age-related muscle atrophy by deacetylation of NF-kB and downregulation of FOXO1, respectively [176,177,178]. AMPK and PGC-1α pathways also play an important role in autophagy in muscle during non-starvation status through the JNK/Bcl-2 anti-apoptotic pathway that dissociates the Beclin1/Bcl-2 complex and elicits autophagy [179].

### 6.3. Molecular Role of Semaglutide in Skeletal Muscle Homeostasis

The interaction between GLP-1 and GLP-1R is observed to present a strong impact on the AMPK signaling within skeletal muscle tissue, augmenting mitochondrial biogenesis, oxidative phosphorylation and metabolism of muscle tissue [180]. Experiments in mice suggest that GLP-1 induces cellular transformations, contributing significantly to enhanced endurance during exercise, thereby underscoring the critical role that GLP-1 plays in the regulation of mitochondrial function and content in skeletal muscle [40].

#### 6.3.1. Semaglutide Improves Obesity-Induced Muscle Atrophy via SIRT1

Semaglutide, in an obesity model in mice, was found to improve skeletal muscle atrophy by activating SIRT1 [181]. In this experiment, in the obese mice group, the expression of muscle atrophy markers Atrogin-1 and MuRF-1 of C2C12 myotube cells increased, and the expression of myogenic differentiation markers (MyoD, Myogenin) and SIRT1 decreased. These parameters significantly improved by the treatment with semaglutide, while the atrophy of myotubes was reversed through SIRT1 activation. Semaglutide also restored impaired glucose tolerance and insulin resistance from the increase in GLUT4 expression also by SIRT1 [181]. In accordance, GLP-1RAs increase microvascular blood flow in muscle tissue, resulting in increased myocyte metabolism, increased muscle mass and muscle atrophy inhibition [182].

#### 6.3.2. Semaglutide Improves Obesity-Induced Muscle Atrophy via Inhibition of UPS Degradation

One other experimental study found that semaglutide inhibited UPS-mediated skeletal muscle proteolysis and degradation, diminishing the ubiquitin ligases atrogin-1 and MuRF-1, and promoted heat-shock factor-1-mediated myogenesis in murine myocytes [183]. Additionally, semaglutide was found to improve skeletal muscle atrophy by directly stimulating GLP-1R signaling in myocytes by the cAMP-mediated activation of PKA and AKT [183]. Semaglutide, also via GLP-1R/cAMP signaling, promoted mitochondrial biogenesis by increasing levels of PGC-1α and SIRT1 and antioxidant activity through the expression of the NRF2-regulated antioxidant genes [183]. These effects were attributable to a GLP-1/GLP-1R axis inhibition of NF-κB, in turn attenuating UPS wasting [183].

## 7. Semaglutide and Anti-Aging

### 7.1. The Crossroad of GLP-1 and Cellular Senescence

Ageing is a time-dynamic process, typically manifested by degeneration of tissue and organ structure and function, cell damage accumulation, dysfunction of cellular organelles and increased susceptibility to diseases [184]. The main mechanisms to foster ageing are multifactorial, including defective mitochondria function and reduction in autophagy activity [185,186].

Aging, obesity and diabetes are co-related [140]. A common crossroad is cellular senescence, a state of indefinite cessation of the cell cycle, triggered by mitotic stress, DNA damage, telomere erosion, mitochondrial dysfunction, oxidative stress, inflammation, mechanical stress and oncogenic activation [187]. Actually, senescence represents the onset of an irreversible cell-cycle withdrawal, and senescent cells, some but not all, develop a senescence-associated secretory phenotype (SASP) [140,184]. SASP represents an aggregation of proinflammatory interleukins, chemokines, growth factors, proteases, receptors, enzymes, toxins, reactive metabolites, bioactive lipids and microRNAs, with the aim to induce immune cells to clear damaged cells but also to prevent oncogenesis [38,188]. However, this senescent cell function wanes with ageing, not only causing dysfunction in body tissues but also triggering inflammation and the formation of cancer cells [140]. Accordingly, SASP directly mediates pancreatic β-cell dysfunction, adipose tissue dysfunction and insulin resistance in peripheral tissues, which promote the onset of T2D and vice versa [189]. In this context, semaglutide may be involved in complex anti-ageing mechanisms like oxidative stress, cellular senescence, chronic inflammation, autophagy and even infection control to lengthen lifespan [184].

### 7.2. Is Semaglutide the Amplifier of Life?

**Figure 3:**
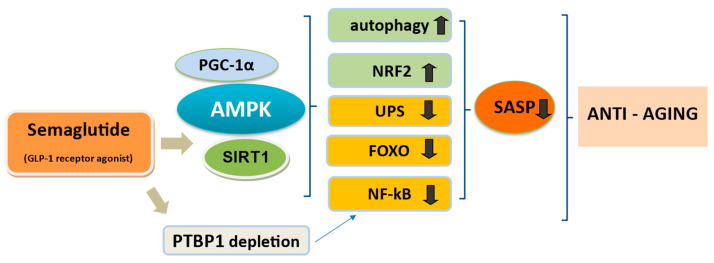
Possible anti-aging mechanisms of semaglutide, hypothesis-generating from preclinical research. Semaglutide through AMPK/SIRT1 activation may blockade the NF-kB critical mediator of inflammation and FOXO transcription factors through SIRT1/PGC-1α. This activates autophagy and may regulate the UPS and autophagy-lysosomal system for removal of senescent cells and SASP. Semaglutide may upregulate transcriptional responses to oxidative stress and ROS production like NRF2 which decrease with ageing. In addition, possible PTBP1 depletion by semaglutide downmodulates the NF-kB pathway. Upward arrows symbolize increase or upregulation, while downward arrows indicate decrease or downregulation of the mentioned mechanisms. Abbreviations: UPS, ubiquitin–proteasome system; SASP, senescence-associated secretory phenotype; PTP1B, protein tyrosine phosphatase 1B or polypyrimidine tract binding protein 1; PGC1a, peroxisome proliferator activated receptor gamma coactivator 1 alpha; FOXO, forkhead box O proteins.

Semaglutide can activate several signaling pathways compromised during ageing, mainly AMPK and SIRT1, which play an important role between autophagy and senescence, engaged in the pathobiology of ageing and age-linked disorders (Figure 3) [190]. NF-kB and C/EBPα complexes, both associated with inflammatory responses, are currently recognized as the main transcription factors globally regulating SASP expression [191]. Semaglutide, i.e., through AMPK/SIRT1/PGC-1α, may blockade NF-kB and FOXO transcription factors, revealed as critical mediators in inflammation of crucial cellular components in mammals during ageing [177,187,192,193]. In addition, PTBP1 depletion by semaglutide downmodulates the NF-kB pathway and mechanistically can be envisioned to counteract the inflammatory effects of SASP [191]. Semaglutide may also upregulate transcriptional responses to oxidative stress, which decrease with ageing, owing to the declining function of the stress-responsive NRF2 [194]. UPS dysregulation occurs in the ageing process and several ageing-related diseases in mammals, and this proteostasis system failure accelerates the accumulation of misfolded proteins and aggregation of damaged organelles [195]. Semaglutide interferes with the UPS and autophagy-lysosomal system, two crucial mechanisms that mediate the renewal of cellular organelles and removal of large aggregates like SASP [182,195]. Many age-related pathologies and the ageing process itself are accompanied by dysregulation of UPS, autophagy and crosstalk between both systems [139].

The protective effects of semaglutide against various toxic stimuli, including high glucose, FFAs, cytokines and ROS, lead to the upregulation of antiapoptotic proteins, such as Bcl-2 and Bcl-xl, and the downregulation of proapoptotic proteins, such as Bcl-2-associated X protein (Bax), Bcl-2 antagonist of death (Bad) and caspases [46,196]. These enhancements might be necessary for lifetime extension and rejuvenation. Nevertheless, because these mechanisms have been demonstrated mostly in short-lived organisms from the yeast model to the mice model [197], from bench to bedside, targeted different interventions to promote longevity, like exercise, nutrient supply reduction and food polyphenols, are optimal to implement [67,153,191,193,198].

## 8. Semaglutide and Infections

Recently, it was found that semaglutide could improve symptoms caused by sepsis and decrease the bacterial load in multiple organs, along with the levels of inflammatory factors in plasma and lungs [199]. This action requires central neuronal GLP-1Rs, of which activation leads to reduced TNF-α via α1-adrenergic, δ-opioid and κ-opioid receptor signaling [199].

Semaglutide may be able to regulate inflammation in various ways. Obesity has been associated with higher gut permeability and subsequent systemic (mild) elevation in circulating plasma LPSs, which are endotoxins found on the cell membranes of Gram-negative bacteria [200]. LPSs are potent activators of the inflammatory response through activation of specific toll-like receptors 4 (TLR4). LPSs induce GLP-1 secretion through this TLR4-dependent pathway when gut barrier function is compromised [19,201]. Activation of the GLP-1 receptor reduces the expression of LPS-induced pro-inflammatory cytokines, including IL-1β, IL-6, TNF-α and IFN-γ, in vivo and in vitro [202]. Other data pose the gut-brain GLP-1R axis in a concept of brain-immune networks for suppression of peripheral inflammation [203]. GLP-1RAs are found to modulate immune cell signaling by cAMP/PI3K-AKT and regulate NF-κB as the crucial inflammation controller [159,204]. Initiating the anti-inflammatory AMPK/SIRT1 pathway, semaglutide may exhibit substantial anti-inflammatory effects and may inhibit the aberrant autophagy caused by ROS in a sepsis model in mice, potentially by reactivating autophagy Beclin-1 pathways [205]. In addition, AMPK, through activation of FOXO1, can regulate transcription factor forkhead box protein 3 (FOXP3), the key gene for differentiation and homeostasis of T regulators lymphocytes (Tregs) presenting immunoregulatory and anti-inflammatory effects [202,206].

## 9. Discussion and Conclusions

In the complex realm of obesity and diabetes semaglutide appears as a promising innovative treatment that could enhance patient outcomes in cardiovascular diseases. Summarizing the evidence on the topic (Table 1), semaglutide may elicit a wide plethora of biological effects from different dimensions. Because of primitive molecular experimental research in humans, semaglutide mechanisms were acquired from reviews and mainly from experimental evidence in rodents. Documented experimental evidence, extracted from important preclinical studies, is described accordingly in Table 2.

As a widely used hypoglycemic drug in clinical practice, semaglutide not only regulates glucose metabolism but presents anti-inflammatory, autophagy-regulating and mitochondrial biogenesis-regulating effects, and it has also been reported to alleviate oxidative stress and improve skeletal muscle mass, all these effects independently of body weight reduction. However, the biological function mediated by semaglutide implicates multiple transcriptional regulators, presenting in some way either promoting or inhibiting activity in a network extremely sensitive to homeostatic imbalance and environmental factors.

Elaborating the results, semaglutide and the GLP-1/GLP-1R signaling system play a pivotal role in glucose regulation by enhancing insulin synthesis though the cAMP-dependent PKA/PI3K pathway, inhibition of pancreatic β-cells apoptosis through AMPK and cAMP-activated CREB and proliferation of pancreatic β-cells by regulation of downstream effector molecules like FOXO1. In addition, semaglutide ameliorates insulin resistance through GLUT4-mediated glucose transport by PI3K/AKT, AMPK-SIRT1 and upregulating phosphorylated IRS-1.

The essential action of semaglutide is the significant beneficial effect in weight loss, directly by interaction with GLP-1 receptors in the central nervous system to activate anorexigenic signaling and to exert an appetite regulation effect. This signaling includes stimulation of anorexigenic melanocortin and POMC/CART neurons and indirect inhibition of orexigenic NPY/AgRP in the hypothalamus. WAT-derived leptin also contributes to anorexigenic effects in the brain. In obesity and T2D, leptin resistance and higher circulating levels drastically reduce leptin signaling in POMC neurons. Experiments in mice showed the interaction of semaglutide with anorexic leptin-sensitive signal transducers and activators like IRS and AMPK/SIRT1/GLUT4 and inhibition of the leptin suppressor PTP1B/SOCS3 pathway to restore leptin. By mobilizing these sophisticated neural circuits within the melanocortin system, semaglutide may regulate energy metabolism and body weight. Because semaglutide and other GLP-1 analogs act in the brain, when taking into account possible adverse effects during GLP-1 treatment, caution may be considered for the influence of semaglutide in the limbic system of the brain during an individualized approach.

Further, semaglutide demonstrated from experimental evidence that it activated adipocyte browning, mainly on subcutaneous fat adipocytes by improving UCP1, mitochondrial biogenesis and thermogenesis, promoting weight loss. Moreover, semaglutide may possibly facilitate the expression of other markers of adipose tissue browning and differentiation towards WAT or BAT, which is optimal for maintaining body weight. These markers may include the central nervous system AMPK-SIRT1 axis and the master transcriptional regulator factor PGC-1α along with CIDEA, PRDM16, FGF21 and FNDC5. Based on one experiment in mice, semaglutide treatment may improve FGF21 sensitivity to mediate beneficial effects in obesity. CIDEA, activated by PRDM16, appears to be crucial to BAT function and activation in interaction with PGC1α. Besides, semaglutide remarkably ameliorates adipose tissue inflammation and oxidative stress mostly via modulating the activity of AMPK and SIRT1 and the deacetylation of the inflammation controller NF-kB. AMPK/SIRT1 induced by semaglutide may also regulate autophagy and mitophagy, increasing transcription factor TFEB and parkin. Eventually, the AMPK/SIRT1 pathway appears foundational for the molecular mechanisms of semaglutide.

Semaglutide and AMPK/SIRT1/PGC-1α signaling may display an important role in autophagy, inflammation, oxidative stress and insulin resistance in skeletal muscle, which are core pathogenetic mechanisms of sarcopenia in obesity. This may occur by direct activation of GLP-1R in skeletal muscle to increase mitochondrial biogenesis and antioxidant activity. Semaglutide may also attenuate muscle atrophy in obesity by inhibition of UPS, the central proteolytic machinery of mammalian cells and the crucial regulators of ubiquitin-mediated skeletal muscle protein degradation, the ligases atrogin-1 and MuRF-1. Fundamental in this process is found SIRT1, which improves skeletal muscle atrophy in obesity by inhibiting the key atrophy genes in skeletal muscles. These actions of semaglutide are described in experiments in obese mice.

Many age-related pathologies and the ageing process itself are accompanied by dysregulation of UPS, autophagy, NF-kB and FOXO transcription factors and crosstalk between the GLP-1/GLP-1R system with AMPK and SIRT1. This reveals a possible key role for semaglutide in reversing the pathophysiology of senescent cell function by reducing SASP and other critical mediators, potentially PTP1B. In the concept of large-scale biological effects, semaglutide may exhibit from experiments in mice substantial anti-inflammatory outcomes in infections and particularly in sepsis from central neuronal GLP-1 effects, regulation of NF-κB, AMPK activation and GLP-1/AMPK FOXP3 immunoregulation.

Consequently, these mechanisms from preclinical evidence may establish an important pathway for understanding and the application of semaglutide to the management of several human diseases.

Despite the highlights on possible semaglutide beneficial action, limitations of this review exist. The ongoing research as discussed is based predominantly on animal models and has not yet been determined or remains unknown in humans. Possibly, this illustrates a difference between biological effects of semaglutide in animals and humans. Moreover, the preclinical data designed specifically on semaglutide potential actions appear limited for most of the described mechanisms. Furthermore, according to research, the current evidence on the molecular action of semaglutide in humans presents as elusive, and the benefits are based on landmark clinical trials. The lacking available robust evidence in humans is a downside to validate the proposed mechanisms, which is critical for translating these findings into clinical practice.

Despite these limitations, the entire field is keenly anticipating novel clinical and experimental data on semaglutide mechanisms. As a paradigm, the field in the application of semaglutide in polymicrobial sepsis and anti-aging appears intriguing. Therefore, new frontiers are essential in human clinical trials to refine and fully underscore the important interplay between semaglutide and molecular mechanisms in the brain, adipose, muscle and other target tissues. A better understanding of the interconnected phenomena will enable the development of novel applications of semaglutide in the spectrum of human health.

## Figures and Tables

**Table 1:** Overview of possible biological actions and molecular mechanisms of semaglutide. The corresponding references are indicated under brackets.

Semaglutide	Biological Effects	Molecular Mechanisms	References
Pancreatic GLP-1 and insulin related actions	Insulin biosynthesis and secretion	cAMP-dependent-PKA/PI3K/mTOR cAMP/PKA/PI3K/GLUT4 transporter	[20,21,40,44,202]
	Improves insulin sensitivity (in adipose tissue and muscles)	AMPK/SIRT1/GLUT4 transporter Upregulation of phosphorylated IRS-1	[44,120,181] [46]
	Stimulates β-cell proliferation Inhibits β-cell apoptosis	PKA/PI3K downstream FOXO1 regulation AMPK, cAMP-activated CREB	[40]
	Inhibits glucagon release		[40,47]
Brain and appetite regulation actions	Appetite and food intake reduction, satiety inducer Increase energy expenditure	Direct stimulation of anorexigenic melanocortin, POMC/CART neurons Indirect inhibition of orexigenic NPY/AgRP	[17,125,131,203] [114,115,116,131]
	Improves leptin sensitivity	GLP-1 interaction with anorexic leptin signaling	[131]
		Inhibition of suppressor PTP1B/SOCS3	[125,129,131]
		Inhibition of PTP1B counteracts leptin resistance	[116]
	Neuroprotection	Hypothalamic AMPK/SIRT1	[80,120,121]
Adipose tissue responce	Enhances BAT activation via transcriptional regulators Enhances WAT browning	Upregulation of AMPK/SIRT1, UCP1, PRDM16, PGC-1α, CIDEA, FGF21, FNDC5	[1,44,71,72,73,74,75,76,77,78,79,80,81,82,83,84,85,86,87,88,89,90,91,92,93,94,95,154]
	Inhibits WAT inflammation	AMPK/SIRT1 NF-κB deacetylation S100a8, S100a9, Cxcl2 inhibition	[22,35,80,204] [110,111]
	WAT reduction	FGF21 up-regulation Lowering ANGPTL4 and LPL activity	[90,112]
Muscle function	Improve skeletal muscle atrophy in obesity	Direct GLP-1R stimulation in myocytes cAMP-mediated PKA/AKT activation	[181,182,183]
	Regulates autophagy	AMPK/SIRT1/PGC-1α activation	[40,83,177,179,180]
		Reduction in FOXO transcription, atrogin1, MuRF1	[170,173,176,182,183]
		Inhibition of NF-κB Inhibition of UPS-mediated skeletal muscle proteolysis	[181,183]
	May inhibit the transition of myoblasts into adipocytes	May inhibit the anti-myogenic S100B	[110,173,174]
Oxidative stress	Ameliorates mitochondrial function Mitigates mitochondrial and ER oxidative stress Regulates excessive ROS	SIRT1/AMPK activation SIRT1/NRF2 signaling ROS reduction SIRT1/AMPK mediated reduction of JNK/NF-kB ROS production and aberrant UPR activation NF-kB downregulation RAGE-RAGE ROS reduction	[4,35,137,152,154] [157,183,204] [89,158,159]
	Activates autophagy And mitophagy	AMPK pathway activation AMPK increase in TFEB and parkin	[157] [77,156]
		Upregulation of antiapoptotic proteins Bcl-2, Bcl-xl Downregulation of proapoptotic Bax, Bad and caspases	[196,197]
Ageing and age-linked disorders	Activation of signaling, compromised during ageing	AMPK/SIRT1/ PGC-1α activation Blockade of NF-kB and FOXO transcription factors	[177,178,187] [192,193]
		May deplete PTBP1 and downmodulate NF-kB pathway to mitigate the inflammatory effects of SASP SASP modulation by targeting the JAK/STAT pathway	[116,191]
		Upregulation of NRF2	[137,194]
		May ameliorate UPS and autophagy-lysosomal system	[139,182,195]
		Upregulation of antiapoptotic proteins Bcl-2 and Bcl-xl Downregulation of proapoptotic Bax, Bad and caspases	[46,196,197]
Infections/ Sepsis	Attenuate polymicrobial inflammation and sepsis-associated detrimental responses Suppress peripheral inflammation	Central neuronal GLP-1 effects controlled by the endogenous opioid system Regulation of NF-κB mediated inflammation control of receptors TLR4 AMPK activation GLP-1/AMPK FOXP3 immunoregulation	[199] [19,159,201,202] [202,205,206]

Abbreviations: CREB, cAMP-response element binding protein; AMPK, adenosine monophosphate–activated protein kinase; SIRT1, sirtuin 1; UPS, ubiquitin–proteasome system; SASP, senescence-associated secretory phenotype; FOXO, forkhead box O proteins; IRS 1, insulin receptor substrate 1,2; JAK, Janus tyrosine kinase; STAT, signal transducer and activator of transcription; POMC, pro-opiomelanocortin neurons; CART, cocaine-and amphetamine-regulated transcript neurons; NPY, neuropeptide Y; AgRP agouti-related peptide; PTP1B, protein tyrosine phosphatase 1B; SOSC3, cytokine signaling 3; UCP1, uncoupling protein 1; PRDM16, PR domain-containing 16; PGC1a, peroxisome proliferator activated receptor gamma coactivator 1 alpha; FGF21, fibroblast growth factor 21; FNDC5, protein fibronectin type III domain-containing protein 5n; CIDEA, cell death-inducing DNA fragmentation factor-α-like effector A; MuRF1, muscle RING-finger protein-1; TFEB, transcription factor EB; ANGPTL4, angiopoietin-like 4; LPL, lipoprotein lipase; TLR4, toll-like receptors 4; FOXP3, transcription factor forkhead box protein 3; Cxcl2, CXC motif chemokine ligand 2; protein arginine methyltransferase-1; Bax, Bcl-2-associated X protein; Bad, Bcl-2 antagonist of death.

**Table 2:** Basic experimental evidence, designed on specification to semaglutide potential mechanisms of action. The corresponding references are indicated under brackets. Abbreviations are found at the end of the table.

Study Ref.	Study Details Type of Mice, Aims, Methods, Interventions	Experimental Procedures	Results	Concluding Points
[46] 2019 Yang et al.	Sprague–Dawley (SD) male rats weighing 250–300 g aged 3 months Aim: Protective effects of semaglutide against middle cerebral artery occlusion injury in rats In vivo experiment: Randomly divided into three groups: Group 1: rats that received equal volume saline (Sham); group2: vehicle controls that received equal volume saline (Vehicle); group 3: semaglutide rats that received semaglutide at 10 nmol/kg i.p. observed for 21 days	Permanent middle cerebral artery occlusion (pMCAO) model for cerebral ischemia Tissue sampling Western blot analysis of the extracted proteins Immunofluorescence staining to detect DCX + cells in the hippocampal dentate gyrus at 1, 7, 14 and 21 days after MCAO	Semaglutide significantly reduced neurological impairments, increased hippocampal neuronal survival post ischemia Semaglutide can re-sensitize insulin signaling and normalize IRS1 activity Semaglutide reduces apoptosis signaling Bcl-2/BAX, Caspase-3	Semaglutide normalize insulin signaling and activity of the IRS1 Reduces apoptosis Neuroprotection
[90] 2022 Feng et al.	Six-week-old male C57BL/6J mice Aim: Effect of semaglutide on FGF21 in high-fat diet mice In vivo experiment: For 13 weeks either fed with low-fat diet (LFD) or HFD HFD randomly divided for daily i.p. semaglutide at high dosage (600 μg/kg) weight) or control PBS injection for 1 week	Plasma: mouse adiponectin immunoassay kit, leptin Tissues: liver, WAT, BAT RT-PCR to determine effects of hFGF21 Quantitative reverse transcription PCR for RNA extraction Western blotting antibodies on prepared whole-cell lysates from liver, adipose to determine effects of hFGF21	In HFD-semaglutide mice: Profound body weight lowering effect possibly inducing a “fasting-like” state in HFD Semaglutide: Reversed hypeleptinemia Elevated FGF21 Stimulated hepatic FGF21 Reduced and reversed HFD alterations in WAT Reduced WAT FGF21 resistance Maintained BAT	Confirmed that semaglutide can upregulate hepatic FGF21 production, restore FGF21 sensitivity, reverse HFD alterations in WAT and maintain BAT
[95] 2022 Martins et al.	C57BL/6 male mice Aim: Evaluation of response of adipocytes to semaglutide In vivo experiment: Two groups (a) control diet (C group) (b) high-fat diet (HF group) for 16 weeks After, each group was randomly Separated into two groups adding semaglutide s.c at 40 μg/kg once every 3 days and studied for an additional four weeks: (a) C, (b) control diet and semaglutide (CS), (c) HF diet, and (d) high-fat diet and semaglutide (HFS) Untreated groups were given sterile s.c saline.	Histology, fat pad fragments UCP1 staining, immunofluorescence Immunohistochemistry qRT-PCR mRNA expression	In obese mice semaglutide: Reduced WAT, proinflammatory markers, leptin (−80%) Lessened ER stress, enhanced UCP1 labeling, PPAR-α (+560%), PPAR-γ (+150%), NRF-1 (+260%) Increased thermogenetic gene expressions for the browning phenotype maintenance: beta-3 adrenergic receptor (+520%), Ucp1 (+110%)	Semaglutide lessens ER stress in WAT Semaglutide mitigates adipocyte hypertrophy Semaglutide enhances browning mediators and mitochondrial biogenesis in WAT
[110] 2024 Pan et al.	Six-week-old C57bl6 mice Aim: Uncover the cardioprotective impact of semaglutide In vivo experiment: Randomly assigned into normal chow diet (NCD), high-fat group (HFD) After 12 weeks, the HFD group was divided into HFD continued to eat high-fat diet and the semaglutide intervention group (Sema group) high-fat diet and semaglutide 30 nmol/kg/day i.p. for 12 weeks	Serum tumor necrosis factor-α (TNF-α), interleukin-6 (IL-6) and malonic dialdehyde (MDA) levels were detected by ELISA Heart tissue ROS content measured by ROS staining of frozen sections Fluorescent microscopy Single-cell RNA sequencing screening for differentially expressed genes (DEGs)	Semaglutide reduced levels of ROS, IL-6, MDA, and TNF in cardiac tissue and serum Semaglutide significantly decreased the highly expressed in neutrophils S100a8, S100a9, and Cxcl2	Semaglutide reduces body weight, improves inflammation and oxidative stress Inhibits of the expression of neutrophil inflammatory factors Whether semaglutide alleviates cardiac inflammation, and oxidative stress independently of body weight reduction; needs further study
[112] 2023 Zhu et al.	C57BL/6JC male mice, 7-week-old, 16–20 g Aim: Role of semaglutide in WAT In vivo experiment: Randomly divided to normal chow diet group (NCD) and high-fat diet (HFD) After 12 weeks of feeding Randomly divided to HFD+saline group (HFD) and HFD+semaglutide i.p. (30 nmol/kg/d) group (HFD-Sema) for 12 weeks	Serum analysis Histopathological analysis of epididymal WAT and interscapular BAT Paraffin blocks for hematoxylin and eosin (H&E) stain Peptide labeling Proteomic analysis Quantitation analysis Bioinformatics	In Semaglutide group vs HFD: (H&E) staining: diameter of adipocytes in WAT and BAT was markedly decreased Proteomics: 640 differentially expressed proteins (DEPs), 292 up-regulated and 348 down-regulated Bioinformatics: reduction of ANGPTL4, LPL	Semaglutide group: Reduction of 10 proteins involved in fatty acid transport and among them, adipogenic ANGPTL4, LPL Significant weight-loss effect Decreased WAT Increased BAT
[120] 2023 Wang et al.	12-month-old male APP/PS1/Tau transgenic mice (3xTg) and C57B6/129 wild type mice (WT) Aim: Effect of semaglutide on glucose metabolism dysfunction in Alzheimer disease (AD) mice and cells In vivo experiment: Randomly assigned to four groups: WT + Saline, 3xTg + Saline, 3xTg + semaglutide, and 3xTg + semaglutide + EX527 (SIRT1 inhibitor) 15 mice in each group were injected with semaglutide (0.1 mg/kg, i. p. or saline (0.9%, i. p.) every other day for 30 days	Micro-PET/CT scanner Immunohistochemistry for brain tissue sections Immunofluorescence with DAPI staining Western blot and protein quantification performed by BCA protein assay Cell immunofluorescence with SIRT1 primary antibody Cell culture of HT22 cells	Semaglutide promoted the expression of SIRT1 and GLUT4 in 3xTg mice Semaglutide alleviated the Aβ and tau pathology in the hippocampus CA3 region of 3xTg mice	Semaglutide reduces mediated by GLP-1/SIRT1/GLUT4 pathway Semaglutide reduces pathology of AD animals
[131] 2023 Martins et al.	DIO male mice (C57BL/6J) Aim: Effect of semaglutide on the neuropeptide signaling implicated in hypothalamic energy metabolism in obede mice In vivo experiment: control diet (C) and high-fat diet (HF) for 16 weeks then re-divided in control diet (C), control plus semaglutides (CS), high-fat (HF), high-fat plus semaglutide (HFS) and high fat pair-feeding groups (HFPF) for 4 weeks	Plasma analysis to determine -active GLP-1 by multiplex biomarker immunoassays -leptin concentration by enzyme immune-assay kit The hypothalamus was stereotaxically sectioned and prepared for biochemical and molecular analysis Immunofluorescence qRT-PCR for hypothalamic mRNA gene expression Confocal laser scanning microscopy and DAPI staining for POMC and NPY labeling	DIO mice showed increased energy intake and body weight linked to leptin resistance Semaglutide: Improved GLP-1 in HFS vs. HF (+500%) Improved leptin in HF vs. C (+95%), but lessened in HFS vs. HF (−46%) Increased *Socs3* in HF vs. C (+300%) but diminished in HFS vs. HF (70%) Augmented *Pomc* in the ARC (HFS vs. HF, +138%) Increased POMC labeling Reduced NPY labeling	Semaglutide treatment restored GLP-1 levels, regulated Socs3 expression in ARC, improved leptin sensitivity and the hypothalamic anorexigenic signaling (POMC/MC4R) for obesity control in DIO mice
[154] 2024 Yan et al.	Six-week-old male C57BL/6J mice Aim: Mechanisms and effects of semaglutide on myocardium injury and cardiac function in diabetic cardiomyopathy mice In vivo experiment: Randomly divided into four groups: control group, semaglutide group, diabetes group and diabetes + semaglutide treatment group Type 1 diabetes induced by i.p. streptozotocin Mice in the semaglutide intervention group were injected s.c semaglutide (0.15 mg/kg) every week for 8 week	Myocardial tissues sampling Western blot analysis RNA isolation and qRT- PCR Immunofluorescence staining Incubation with the primary antibody Cx43, nuclei were stained with DAPI	In the semaglutide-treated group when compared to the untreated: Semaglutide Significantly increased antioxidant enzymes (SOD) Significantly increased SIRT1/AMPK Normalized Cx43 protein expression Reversed RR, QRS, QT, and QTc intervals prolongation in ECG	SIRT1/AMPK signaling pathways may mediate the cardioprotective effects of semaglutide
[157] 2020 Li et al.	Male 4–6 weeks old SD rats, weighting 200–250 g Aim: Effects and mechanism of semaglutide on exercise-induced myocardial injury In vivo experiment: Divided randomly into normal rat group and overtraining rats total training 10 weeks then overtraining rats randomly divided as high dose of semaglutide treated group, medium dose semaglutide treated group, low dose semaglutide treated group and control rats without semaglutide treatment for 8-week	Cardiac tissues and blood samples LPS-induced oxidative stress injuries and inflammatory Response, assessed in H9c2 cell via MTT assay and Western blot Protein preparations, primary antibodies, H9c2 embryonic rat heart-derived (ventricular) cells Apoptosis detection kit and ROS detection kit	Semaglutide improve the viability and apoptosis of LPS treated myocardial H9C2 cells Semaglutide activate AMPK pathway, improve autophagy and inhibit ROS production in LPS treated H9C2 cells Semaglutide ameliorates myocardial injury markers in excessive exercise rat model	Semaglutide may reduce the inflammatory response by activating the AMPK pathway, inhibiting oxidative stress (ROS), improve autophagy and downregulate inflammatory cytokines (NF-kB)
[181] 2023 Xiang et al.	Male C57BL/6 mice (8 weeks old) weighing 20.1 ± 1.1 g Aim: Effect and molecular mechanisms of GLP-1R agonists liraglutide and semaglutide on obesity-induced muscle atrophy In vivo experiment: Mice randomly divided into regular diet and a high-fat diet group for 18 weeks After modeling obesity, mice were further divided into control group, liraglutide (LIRA) group, semaglutide (SEMA) group, high-fat diet (HFD) group, HFD + LIRA group, HFD + SEMA group, Semaglutide (60 ug/kg/d) s.c for 4 weeks	Histological analysis of gastrocnemius muscle C2C12 cells culture C2C12 myotubes were incubated with palmitic acid to induce obesity and skeletal muscle atrophy Immunofluorescence, nuclei were stained with DAPI qRT-PCR Western Blotting	HFD up-regulated the expression of muscle atrophy factor Atrogin-1 and suppressed the expression of myogenic factor Myogenin and SIRT1 Semaglutide: Activated SIRT1 Reduced atrogin-1 expression Increased myogenin expression Alleviated the decrease in GLUT4 expression induced by HFD	Semaglutide activation of SIRT-1 may be important to ameliorate insulin resistance upregulating GLUT4 and to alleviate obesity induced muscle atrophy
[183] 2023 Iwai et al.	Male diabetic KK-Ay mice aged 10 weeks old Aim: Effect of semaglutide on skeletal muscle wasting and dysfunction in liver disease-related skeletal muscle atrophy under diabetic conditions In vivo experiment: four groups treated for six weeks as follows: (i) normal diet (ND-Veh) (ii) ND and s.c.semaglutide (3 nmol/kg) every 3 days (ND-Sem group) (iii) DDC diet (iv) DDC diet plus s.c semaglutide every three days (DDC-Sem group) Vehicle: saline	Histological and immunofluorescent analyses of gastrocnemius muscle tissue RNA isolation and qRT-PCR Western blotting assay gel electrophoresis and primary antibodies incubation Tissue and whole cell lysates were extracted from gastrocnemius muscle tissues Cultured C2C12 myotubes Antibodies for western blotting were atrogin-1, MuRF1, myogenin, PGC-1α, SIRT1	No significant differences in body length or weight between the ND-Veh and ND-Sem groups for six weeks treatment Intramuscular atrogin-1 and MuRF-1 levels increased in the DDC-Veh group, with this difference significantly attenuated in the DDC-Semaglutide group Semaglutide increased PGC-1α, SIRT1 Reduced NF-kB	Limited effect of semaglutide on physiological status Semaglutide: Directly activates GLP-1R in skeletal muscle to increase mitochondrial biogenesis and antioxidant activity Inhibits UPS Suppresses protein degradation Promotes myogenesis in mice Improve skeletal muscle atrophy in obesity

Abbreviations: DIO, diet-induced obesity mice; DDC, diethoxycarbonyl-1,4- dihydrocollidine diet; qRT-PCR, real-time quantitative reverse transcription PCR; s.c., subcutaneous injection; i.p, intraperitoneal; FGF21, fibroblast growth factor 21; LPS, lipopolysaccharide; ER, endoplasmic reticulum; NRF-1, nuclear respiratory factor 1; UCP1, uncoupled protein 1; SOD, superoxide dismutase; POMC, proopiomelanocortin; MC4R, melanocortin-4-receptor; ARC, arcuate nucleus; MuRF1, muscle RING-finger protein-1; UPS, ubiquitin–proteasome system.
